# Treatise on Uterine Displacement

**Published:** 1853-07

**Authors:** 


*Treatise on Uterine Displacements.* By WM. EWD. COALE, M. D. Boston, 1852.

THIS unpretending little pamphlet contains more really useful information on the subject of uterine displacements, than many larger and more pompous treatises. It is plain, practical and to the point. The examination of the causes of prolapsus is especially valuable, as pointing out a gross violation of hygienic rules in the present mode of dressing among our women. The physiological positions of our author are clearly stated and ably defended. No anatomist can fail to be convinced by them.

To those who wish a practical text-book on this important class of diseases, we strongly recommend this little pamphlet of 52 pages.

